# Computerized Working-Memory Training as a Candidate Adjunctive Treatment for Addiction

**DOI:** 10.35946/arcr.v36.1.12

**Published:** 2014

**Authors:** Warren K. Bickel, Lara Moody, Amanda Quisenberry

**Affiliations:** Warren K. Bickel, Ph.D., is the director for the Addiction Recovery Research Center at the Virginia Tech Carilion Research Institute and a professor in the Department of Psychology at Virginia Tech, Roanoke, Virginia. Lara Moody is a clinical psychology graduate student at Virginia Tech and a graduate research assistant in the Addiction Recovery Research Center at the Virginia Tech Carilion Research Institute, Roanoke, Virginia. Amanda Quisenberry, Ph.D., is a postdoctoral associate at the Virginia Tech Carilion Research Institute, Roanoke, Virginia.

**Keywords:** Alcohol and other drug use, abuse, and dependence, alcoholism, addiction, treatment, brain function, working memory, computerized working-memory training, computer technology, electronic health technology

## Abstract

Alcohol and other drug dependencies are, in part, characterized by deficits in executive functioning, including working memory. Working-memory training is a candidate computerized adjunctive intervention for the treatment of alcoholism and other drug dependencies. This article reviews emerging evidence for computerized working memory training as an efficacious adjunctive treatment for drug dependence and highlights future challenges and opportunities in the field of working-memory training, including duration of training needed, persistence of improvements and utility of booster sessions, and selection of patients based on degree of deficits.

Computerized adjuncts for the treatment of alcohol dependence and other drug dependencies have taken many forms (Bickel et al. 2011). Some have focused on computerizing various forms of cognitive–behavior therapy (CBT) (Bickel et al. 2008; Budney et al. 2007; Carroll et al. 2004). Other approaches have focused on rehabilitating aspects of executive dysfunction (Bates et al. 2013), or as it has been called in other literatures, impulsivity (Bickel et al. 2012). The importance of the latter foci is supported by evidence that between 50 and 80 percent of people with alcohol disorders or other drug dependencies experience mild to severe executive function impairments (Aharonovich et al. 2006; Bates et al. 2006; Goldman 1990; Gonzalez et al. 2004). Among people in substance abuse treatment, these neuropsychological impairments are related to greater attrition, violations of clinic rules, and poor treatment outcomes (Aharonovich et al. 2003, 2006; Bates et al. 2006; Teichner et al. 2002).

Rehabilitative efforts focused on improving executive function have been increasing in the last 10 years, partly as a result of advances in computerized training, particularly “adaptive-training” programs (Klingberg 2010). Adaptive-training programs rely on computerized algorithms that adjust intervention content to a patient’s skill level in real-time in order to tax participants at the limit of their capacity and maintain engagement during training (Morrison and Chein 2011). Other advantages of computerized training include standardized delivery of intervention content and the ability to automatically track a patient’s progress in relation to the dose, duration, and content of the training received (Bickel et al. 2011*a*). The increasing reach of computer technology and the Internet, which can provide patients with greater access to adjunctive interventions at times and places that fit their schedule, also contributes to interest in computerized training programs (Bickel et al. 2011*a*).

Computerized training to address executive function has focused on broad-based training or training of specific executive functions (Bates et al. 2013). One such computerized approach has trained a form of response inhibition to certain alcohol-or drug-related stimuli (e.g., attention bias modification [Wiers et al. 2013]) as a means to prevent the automaticity often observed in addiction. This article will focus on another computerized approach that trains a specific executive function, namely, working memory.

Working memory refers to “the ability to retain some information active for further use, and to do so in a flexible way allowing information to be prioritized, added, or removed” (Bledowski et al. 2010, p. 172). Some investigators have suggested that working-memory is a foundational executive function that undergirds many others (Baddeley 2012). In addition, Hofmann and colleagues (2012) have suggested that working-memory operations undergird successful self-regulation. More specifically, they state that working memory is important for (1) adequate representation of self-regulatory goals, (2) the control of attention, and (3) protecting goals from interferences such as desires and craving. Thus, working-memory capacity may be related to delay discounting, which refers to the discounting of the value of a reward as a function of longer delays to receipt of the reward (Bickel et al. 2011*a*). Specifically, people with lower working-memory capacity may show greater delay discounting, a form of impulsivity, by preferring sooner, smaller rewards relative to later, larger rewards (Bickel et al. 2011).

Not surprisingly, working-memory deficits and excessive delay discounting have been observed in substance-dependent groups, including alcohol-(e.g., Beatty et al. 1995), cocaine-(e.g., Berry et al. 1993), methamphetamine(e.g., McKetin and Mattick 1997; Bickel et al. 2011*a*) and opioid-dependent individuals (e.g., Ersche et al. 2006). Of course, these groups typically show other executive dysfunctions. However, if working memory is central to the other executive functions, theoretically related to self-regulation and delay discounting, and diminished or dysfunctional among those with alcohol or other drug dependencies, then examining the effects of working-memory training in addiction is worthwhile.

This article will review the current research on the use of computerized working-memory training as a target for intervention in addiction. Specifically, it will (1) review the status of working-memory training as a relevant tool in addiction treatment, and (2) address potential challenges and opportunities related to the use of working-memory training as an adjunctive treatment.

## Computerized Working-Memory Training

Working-memory training has been identified as a possible means to enhance executive function in various populations (for a review, see Shipstead et al. 2012). Typically, computerized working-memory training occurs several times a week, over multiple weeks (e.g., 4 to 6 weeks), during which four to eight blocks of working-memory tasks are completed (Klingberg 2010). Computerized training programs have been developed to address different aspects of working memory, have been administered according to various schedules and durations in different populations, and have demonstrated mixed findings with regard to generalizability and sustainability of training effects (Klingberg 2010; Shipstead et al. 2012).

Within healthy populations, evidence of working-memory improvement has been limited and often conflicting. For example, in young adults, two studies (Jaeggi et al. 2008, 2010) found that computerized working-memory training was not associated with increases in fluid intelligence, although a third study using the same working-memory training exercise (i.e., dual N-back^1^) did find this improvement in older adults (Seidler et al. 2010). One possible explanation for these contradictory results throughout the healthy population literature (for a review, see Shipstead et al. 2012) is that improvements are harder to achieve or less consistent in those with adequate abilities. Thus, improvements may have only been seen in people with significant deficits, such as elderly people, people with schizophrenia, and those with alcohol or drug dependencies (Lett et al. 2014).

Studies of working memory and other executive-function training within impaired populations have proved more promising. Bickel and colleagues (2011*b*) examined effects pre– and post–working-memory training in an experimental and control group of stimulant-dependent individuals in treatment. In the experimental condition, participants completed a series of computerized working-memory tasks, whereas those in the control group received a similar task battery where the answers were provided so that working-memory ability was not taxed. After receiving between 4 and 15 training sessions, the excessive-delay discounting of the experimental group decreased significantly more than in the control group, suggesting increased self-control and valuation of delayed rewards in the experimental group. Changes in delay discounting were not accompanied by changes in other measures that were concurrently assessed, including a response inhibition task. One possible explanation is that the neural areas associated with working memory and future valuation (i.e., delay discounting) overlap in the posterior dorsolateral prefrontal cortices, which would support concurrent change in working memory and future valuation but not change in behaviors subserved by brain regions/circuits with less overlap (Wesley and Bickel 2014).

Another study of working-memory training conducted via the Internet in problem drinkers found reduced alcohol consumption, particularly in more impulsive individuals (Houben et al. 2011). Houben and colleagues (2011) provided problem drinkers with 25 sessions of either active or control working-memory training. At the conclusion of computerized training, working-memory improvements and decreased alcohol intake were demonstrated in the experimental group and persisted 1 month after training cessation. Moreover, they found that people with stronger automatic (implicit) preferences for alcohol benefited the most from working-memory training. The reduction in discounting of future rewards in stimulant users (Bickel et al. 2011*b*) and the finding of reduced alcohol consumption in problem drinkers (Houben et al. 2011) provide converging evidence that suggests computerized working-memory training can improve working memory, aspects of self-regulation (e.g., delay discounting), and excessive alcohol consumption in certain subgroups (see Verbeken et al. 2013 for interesting complementary findings in obesity treatment).

The exact mechanism or mechanisms of these effects are unknown. One mechanism may be related to a conceptual model of addiction that stipulates an imbalance between two neurobehavioral decision systems that should ideally be in regulatory balance (Bechara and Damasio 2002; Bickel et al. 2014; Jentsch and Taylor 1999). This model is a specialized variant of the numerous dual models developed to address nonpathological behavior (Kahneman and Tversky 1979). In the addiction-related dual model, referred to as the competing neurobehavioral decision systems hypothesis, individuals with addiction often show greater control by the impulsive decision system and less by the executive decision system. The impulsive decision system is embodied in the limbic and paralimbic brain regions and often functions in the short term to obtain biologically relevant reinforcers. The executive decision system is embodied in aspects of the prefrontal cortices and functions to obtain longer-term outcomes and reinforcers. Working-memory training, by strengthening an aspect of the executive decision system, may reestablish some degree of regulatory balance in addicted individuals.

## Conclusions: The Challenges and Opportunities of Working-Memory Training

Working-memory training is not a panacea, but for some individuals receiving treatment for alcohol or other drug dependencies it may be a useful adjunct. Some of the challenges and opportunities related to working-memory training are reviewed below.

Challenges of computerized working-memory training are at least sixfold. First, the number and breadth of working-memory training sessions necessary to produce an improvement on working memory or other outcomes like delay discounting (a measure of impulsivity or self-control) are unknown. Second, it is not known whether the extent of training would vary by the type of drug dependence or the degree of dependence. A recent study (Bickel et al. 2014) suggests that the largest effect of working-memory training will occur among those who discounted delayed rewards the most at the start of treatment. Third, the duration of the improvements in working memory once trained also is unknown. However, two studies with clinical populations have shown sustained effects of working-memory training from 1 month (Houben et al. 2011) to 2 months (Verbeken et al. 2013). Fourth, if the effect dissipates, research is needed to determine whether booster sessions of working-memory training could facilitate retention of the clinical improvements. Fifth, working-memory training can be long and laborious for the participant, raising questions about motivation techniques that would ensure compliance with the training regimen. Sixth and finally, the extent to which working-memory training will generalize to behaviors beyond alcohol consumption and delay discounting remains to be determined.

The opportunities associated with computerized working-memory training lie in its potential to improve the efficacy of existing treatments as an adjunctive intervention. If patients at the beginning of treatment could complete an assessment that would discern their working-memory ability or perhaps their delay discounting, then those individuals showing the greatest impairment could receive adjunctive treatment with computerized working-memory training (Bickel et al. 2014; McCrady and Smith 1986). Whether this training should occur concurrently with other aspects of treatment or start before the other treatment components is an important issue to address. That is, those patients with executive dysfunction may not be able to benefit from important aspects of treatment until some of their dysfunction has been repaired. Nonetheless, the prevalence of working-memory dysfunction in alcohol and other drug dependencies, its relationship to poor clinical outcomes, and the theoretical relationship between working memory ability and self-regulation collectively suggest the importance of exploring the full therapeutic implications of computerized working-memory training as an adjunctive intervention in addictions treatment.

## Footnotes

1 Games based on N-back tests require players to remember the location of a symbol or the sound of a particular letter presented just before (1-back), the time before last (2-back), the time before that (3-back), and so on.
